# Left atrial appendage sealing performance of the Amplatzer Amulet and Watchman FLX device

**DOI:** 10.1007/s10840-022-01336-4

**Published:** 2022-08-11

**Authors:** Kasper Korsholm, Anders Kramer, Asger Andersen, Jacqueline Saw, Bjarne Linde Nørgaard, Jesper Møller Jensen, Jens Erik Nielsen-Kudsk

**Affiliations:** 1 Department of Cardiology, Aarhus University Hospital, Palle Juul-Jensens, Boulevard 99, 8200 Aarhus, Denmark; 2 Department of Cardiology, Vancouver General Hospital, Vancouver, Canada

**Keywords:** Left atrial appendage occlusion, Watchman, Amulet, Atrial fibrillation, Computed tomography

## Abstract

**Background:**

The left atrial appendage (LAA) sealing properties of the Amplatzer Amulet and Watchman FLX devices were compared using cardiac computed tomography (CT) follow-up.

**Methods:**

Single-center cohort study of patients undergoing LAAO between 2017 and 2020. Two consecutive cohorts were enrolled, one treated with the Amplatzer Amulet (*n* = 150) up till 2019, and a second cohort treated with the Watchman FLX (*n* = 150) device from 2019. Cardiac CT was performed 2 months postprocedure. The primary outcome was complete LAA occlusion defined as no visible peri-device leak (PDL) and absence of contrast patency in the distal LAA. Secondary outcomes included PDL, contrast patency without visible PDL, PDL area, and periprocedural complications.

**Results:**

Complete occlusion was achieved in 39 (30.5%) of the Amulet group, compared to 89 (71.8%) of the FLX group, *p* < 0.001. A PDL at the Amulet disc was present in 65 (50.8%), at the lobe in 16 (12.5%), and at both the disc and lobe in 13 (10.2%). For FLX, a PDL was present in 20 (16.1%). Contrast patency without visible PDL was observed in 24 (18.8%) and 15 (12.1%) of the Amulet and FLX group, respectively. The PDL area at the Amulet mid-lobe was 92 mm^2^ (59–158) and 32 mm^2^ (IQR 28–96) for FLX, *p* = 0.019. Device-related thrombosis occurred in 1 (0.7%) and 2 (1.3%), respectively (*p* = 0.99), with periprocedural adverse events occurring in 6 (4%) and 8 (5.3%) of the Amulet and FLX group (*p* = 0.79).

**Conclusion:**

Complete LAA occlusion was achieved in a significantly higher proportion treated with the Watchman FLX compared to the Amulet device. PDL was smaller with the FLX than the Amulet. Conceptual device design differences make interpretation of results complex, and additional studies with clinical outcomes are needed.

## Introduction

Left atrial appendage occlusion (LAAO) represents an alternative to oral anticoagulation for stroke prevention in selected patients with atrial fibrillation (AF) (1). The efficacy and safety have been demonstrated in 3 randomized trials (2, 3) and ongoing trials (4) are assessing the efficacy and safety in various AF populations. Nevertheless, certain issues with current LAAO technologies remain open, such as the incidence of peridevice leak (PDL) and associated risk of thromboembolic events.

PDL is frequently detected during postprocedural device surveillance, with varying frequency depending on the applied imaging modality and implanted LAAO device (5). Up to 30% have PDL at 45-day transesophageal echocardiography (TEE), while cardiac computed tomography (CT) appears twice as sensitive, reporting PDL in up to 60% of the patients (5, 6).

The two most widely used LAAO technologies are developed on the Amplatzer and Watchman platforms, with the contemporary Amulet and FLX devices representing second-generation iterations. The main improvements with the recent Watchman FLX device were increased device conformability and left atrial appendage (LAA) sealing compared to its predecessor, the Watchman 2.5 (7–9). The recent AMULET-IDE (10) and SWISS-APERO (11) trial compared LAA sealing with the AMULET and Watchman devices. The studies were limited by utilization of the non-contemporary Watchman 2.5 device as comparator. The SWISS-APERO trial did mainly include Watchman FLX, but still, 23% of device implants were the Watchman 2.5 generation. This likely confounds results in favor of the Amulet device. The PINNACLE-FLX study reported a remarkably low frequency of PDL with the FLX device using core lab adjudication (9). This has been confirmed by consistent observational studies (7, 8). Hence, data are conflicting and the present study was carried out to compare LAA sealing performance of the contemporary Amplatzer Amulet and Watchman FLX devices based on a single-center experience utilizing cardiac CT follow-up.

## Methods

**Fig. 1:**
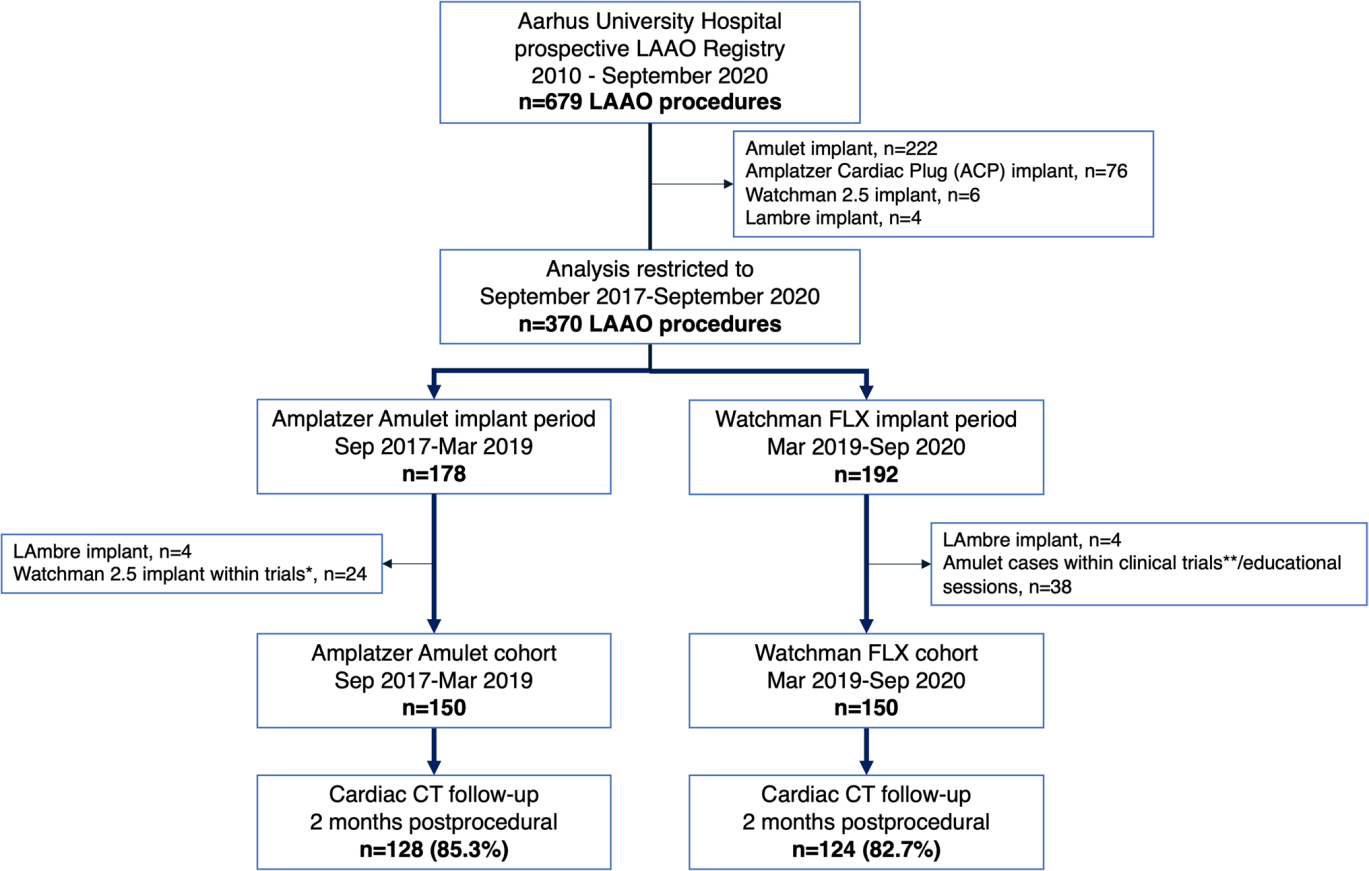
Patient flow chart. *LAAO performed in trials restricted to Watchman 2.5 use. **LAAO closure performed in clinical trials or educational sessions restricted to Amulet device use. Patient enrolment or inclusion in educational sessions were prior to preprocedural CT acquisition and knowledge of the LAA anatomy. LAAO, left atrial appendage occlusion

A single-center, retrospective cohort study was based on consecutive patients undergoing LAAO at Aarhus University Hospital in the period 2017 till 2020. Two operators performed all procedures. The LAAO program was initiated in 2010 using Amplatzer devices. At time of data capture, the local LAAO registry included 679 LAAO procedures (Fig. 1), with 412 (60%) Amulet device implants, 150 (22%) Watchman FLX implants, 76 (11%) Amplatzer Cardiac Plug implants, 30 (4%) Watchman 2.5 implants, and 12 (2%) LAmbre implants. The Amplatzer Amulet cohort was sampled between September 2017 and March 2019. From here, we transitioned to implant Watchman FLX devices in the following cohort till September 2020 (Fig. 1). During the Amulet sampling period, 28 patients received a non-Amulet device due to inclusion in clinical studies restricted to certain devices. In the FLX sampling period, 42 patients received non-FLX devices due to enrolment in clinical trials or an educational session restricted to a specific device. Patient enrolment into trials or educational sessions were prior to acquisition of preprocedural cardiac CT and knowledge of LAA anatomy (Fig. 1).

All baseline, procedural, and follow-up clinical events were prospectively collected in a dedicated LAAO registry. As per institutional practice, all patients underwent preprocedural and 2 months postprocedural cardiac CT evaluation. Cardiac CT was omitted in patients with glomerular filtration rate < 30 ml/min.

The study was approved by the Central Denmark Region (1–45-70–60-20) and performed in accordance with the Helsinki declaration. All patients provided consent prior to intervention.

### LAAO procedures

Preprocedural planning was performed by cardiac CT evaluation, as previously described in detail (12). The procedural setup has been described in full detail for both the Amplatzer Amulet and Watchman FLX implantations (7, 13). Briefly, procedures were carried out in local anesthesia with guidance from intracardiac echocardiography from the left atrium (ViewFlex Xtra, Abbott) and fluoroscopy. An ultrasound-guided double femoral vein access was obtained by a medial 9F 20-cm sheath for the ICE catheter, and a lateral 8.5F sheath for the transseptal system, and later the device delivery system. An inferoposterior transseptal puncture was targeted. After transseptal crossing with a guidewire, the ICE catheter was advanced along the guidewire into the left atrium. The device delivery sheath was then positioned in the left upper pulmonary vein (LUPV), and with a pigtail catheter in front withdrawn into the LA for insertion into the LAA. A selective LAA contrast angiogram was acquired to confirm anatomy and preprocedural device sizing. The LAAO device was deployed under fluoroscopy and ICE guidance according to device specific instructions for use. Final device position, anchoring, compression, and sealing were confirmed by ICE utilizing the LUPV, mid left atrial, and supra-mitral view along with a control contrast angiogram. Procedures were finalized by a figure-8-suture at the access-site for hemostasis.

### Cardiac CT acquisition

Cardiac CT images were acquired using the Siemens Somatom Definition Force scanner (Siemens Healthcare, Forchheim, Germany). The detailed acquisition protocol has previously been described (12). It included a prospective ECG-gated high-pitch single-heart beat spiral acquisition (Flash) using automated tube current modulation (CareDose 4D) and tube voltage set between 70 and 140 kV depending on body weight. A diastolic phase was targeted in heart rates below 70 beats per minute, and a systolic phase in heart rates above 70 beats per minute. Following a test bolus, a single contrast injection (350 mg I/ml iodine concentration) of 40–60 ml was administered through an antecubital vein at flow rates of 5–6 ml/s, followed by a 50-ml saline chaser. Images were reconstructed with a 0.75 mm slice thickness and a medium soft Bv40 kernel.

### Cardiac CT interpretation

**Fig. 2:**
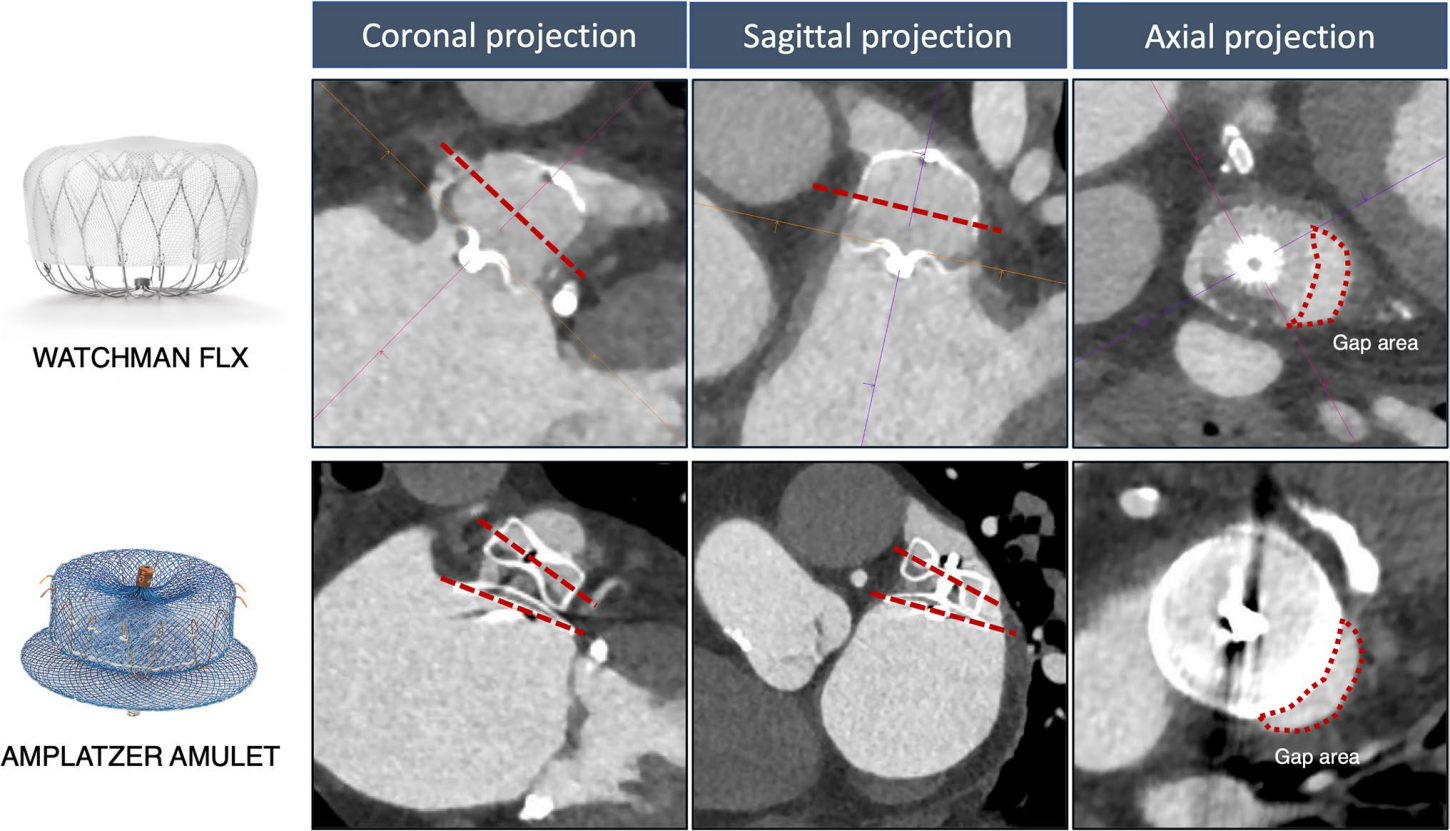
Cardiac CT multiplanar reconstructed views for investigation of peridevice leak. The dotted lines represent the proximal and mid-device cross-sectional views (Watchman FLX device, top panel) and disc and mid-lobe cross-sectional views (Amplatzer Amulet device, bottom panel)

Cardiac CT images were analyzed using syngo.via (Siemens Healthcare, Forchheim, Germany). The implanted LAAO device was identified in the multiplanar reconstructed view (5, 14). Device specific methodology for PDL detection and classification by cardiac CT are described below and in the Fig. 2.

Amplatzer Amulet.

The multiplanar axes were aligned with the disc and the center placed perpendicular through the screw hub (Fig. 2). At the level of the disc, any contrast gap adjacent to the device disc was interpreted as a PDL and the area was measured. A similar approach was applied at the proximal, mid and distal cross-sectional view of the device lobe. A PDL present at all three levels along the device lobe was classified as a device lobe PDL.

Watchman FLX.

The axes were aligned through the device screw hub and the center placed perpendicular through the screw hub (Fig. 2). Any contrast gap adjacent to the device was interpreted as a PDL and the area was measured. A similar approach was repeated at the mid portion of the device. A PDL present at both the proximal and mid portion of the device was classified as a device PDL.

For both devices, contrast patency in the distal LAA was assessed by the Hounsfield attenuation (HU) value, and a reference was measured at the center of the left atrium. A LAA to left atrium (LAA:LA) HU ratio was calculated. Absence of LAA contrast patency was defined as HU < 100 in the distal LAA, or a LAA:LA ratio < 0.25. PDL was graded according to the classification in Table 1.

**Table 1:** Cardiac computed tomography classification of PDL

	**Amplatzer Amulet**			**Watchman FLX**	
	**PDL at disc**	**PDL device lobe**	**LAA contrast patency**	**PDL at lobe**	**LAA contrast patency**
**Complete occlusion**	**-**	**-**	**-**	**-**	**-**
**Grade 1 leak**	**-**	**-**	** + **	**-**	** + **
**Grade 2 leak**	** + **	**-**	** ± **	**NA**	**NA**
**Grade 3 leak**	** + **	** + **	** + **	** + **	** + **

A grade 1–2 leak with the Amplatzer Amulet was regarded comparable to a grade 1 leak with the Watchman FLX device. *PDL* peridevice leak, *LAA* left atrial appendage

### Outcomes

The primary study outcome was complete LAA occlusion defined as no visible PDL and absence of distal LAA contrast patency assessed by cardiac CT. Secondary outcomes included LAA contrast patency, PDL, contrast patency in the distal LAA without visible PDL, PDL area at the level of the disc and mid-lobe of the Amulet, and at the midportion of the FLX device. Major adverse events during the first 30 days following LAAO included stroke, systemic embolism, major bleeding, pericardial effusion requiring drainage, device embolization, and all-cause mortality. Clinical outcomes were defined according to the Munich consensus paper on definitions and endpoints (15).

### Statistics

Data distribution was assessed using QQ-plots and histograms. Continuous variables were expressed as mean with standard deviation (SD), or median with interquartile range (IQR), and were compared using the Student *t*-test or Mann–Whitney *U* test as appropriate. Categorical variables were reported as counts and percentages, and groups were compared using Fishers exact test.

The primary outcome analysis was based on a logistic regression model with a priori adjustment for known predictors of PDL: LAA landing zone diameter, LAA morphology, and permanent atrial fibrillation. A 2-tailed *p*-value < 0.05 was considered statistically significant. Statistical analysis was performed using STATA (STATA IC, version 14.2, StataCorp, College Station, TX).

Design differences make direct head-to-head comparison of PDL complex. A prespecified modified primary outcome analysis was included to account for a few prior studies that considered an isolated PDL at the Amulet disc with a sealing lobe and no LAA contrast patency as a complete LAA occlusion (6, 16). Furthermore, the primary outcome was assessed across implant tertiles in each device group to assess operator learning curve.

## Results

A total of 300 patients were included, 150 patients in each device group. The baseline clinical characteristics between cohorts were balanced (Table 2); however, chicken wing anatomy was more frequent in the Amplatzer Amulet group (38 vs 23%, *p* = 0.008), whereas cactus anatomy was more common in the Watchman FLX cohort (18 vs 9%, *p* = 0.026). The LAA size was comparable, but implanted device was slightly larger in the FLX cohort (Table 3).

**Table 2:** Baseline characteristics

	Amplatzer Amulet	Watchman FLX	*p*-value
	*n* = 150	*n* = 150	
Age at LAAO, mean (SD)	73.4 (9.6)	74.2 (8.3)	0.45
Female	47 (31.3%)	35 (23.3%)	0.15
Body mass index, mean (SD)	27.5 (5.2)	27.2 (4.7)	0.55
Permanent atrial fibrillation	60 (40.0%)	71 (47.3%)	0.24
Congestive heart failure	19 (12.7%)	28 (18.7%)	0.20
LVEF, median (IQR)	60.0 (50.0, 60.0)	60.0 (50.0, 60.0)	0.96
Hypertension	107 (71.3%)	113 (75.3%)	0.51
Diabetes mellitus	28 (18.7%)	36 (24.0%)	0.32
Ischaemic heart disease	50 (33.3%)	52 (34.7%)	0.90
Stroke/TIA/Thrombo-embolism	59 (39.3%)	66 (44.0%)	0.48
Prior bleeding	95 (63.3%)	95 (63.3%)	1.00
Estimated GFR, median (IQR)	77.0 (54.0, 87.0)	72.0 (52.0, 87.0)	0.95
Vascular disease	57 (38.0%)	62 (41.3%)	0.64
Abnormal liver function	3 (2.0%)	2 (1.3%)	1.00
Alcohol	15 (10.0%)	18 (12.0%)	0.71
Renal insufficiency	22 (14.7%)	20 (13.3%)	0.87
Dialysis	5 (3.3%)	5 (3.3%)	1.00
CHA2DS2-VASc score, mean (SD)	3.9 (1.5)	4.1 (1.6)	0.31
HAS-BLED score, mean (SD)	2.7 (1.0)	2.5 (1.0)	0.05
**Indication for LAAO**			
History of intracranial bleeding	28 (18.7%)	28 (18.7%)	1.00
History of GI bleeding	28 (18.7%)	43 (28.7%)	0.06
History of spontaneous bleeding	36 (24.0%)	32 (21.3%)	0.68
High HAS-BLED (≥ 3)	36 (24.0%)	24 (16.0%)	1.00
Stroke despite (N)OAC	13 (8.7%)	12 (8.0%)	1.00
Cerebral amyloid angiopathy	7 (4.7%)	5 (3.3%)	0.77
Other	2 (1.3%)	6 (4.0%)	0.31
**Preprocedural LAA anatomy**			
Chicken wing morphology	56 (37.3%)	35 (23.3%)	0.01
Cactus morphology	14 (9.3%)	27 (18.0%)	0.04
Windsock morphology	69 (46.0%)	75 (50.0%)	0.56
Cauliflower morphology	8 (5.3%)	12 (8.0%)	0.49
Max diameter landing zone, median (IQR)	20.5 (17.0, 27.0)	23.0 (20.0, 26.0)	0.19
Min diameter landing zone, median (IQR)	18.0 (16.0, 22.0)	20.0 (17.0, 24.0)	0.13

Data are presented as mean (SD), median (IQR), or *n* (%)

*LAAO* left atrial appendage occlusion, *LVEF* left ventricular ejection fraction, *TIA* transient ischemic attack, *GFR* glomerular filtration rate, *GI* gastrointestinal, *(N)OAC* (novel) oral anticoagulation

**Table 3:** Procedural characteristics

	Amplatzer Amulet	Watchman FLX	*p*-value
	*n* = 150	*n* = 150	
Imaging guidance			0.089
Transesophageal echo	16 (10.7%)	8 (5.3%)	
Intracardiac echo from LA	134 (89.3%)	142 (94.7%)	
Technical success	150 (100.0%)	150 (100.0%)	1.00
Device size, mean (SD)	25 (4)	28 (4)	< 0.001
First device implanted	140 (93.3%)	143 (95.3%)	0.73
Repositioning attempts			0.67
0	139 (92.7%)	141 (94.0%)	
1	10 (6.7%)	7 (4.7%)	
2	1 (0.7%)	1 (0.7%)	
3	0 (0.0%)	1 (0.7%)	
Procedure time, median (IQR)	36 (27, 46)	37 (32, 45)	0.29
Fluoroscopy time, median (IQR)	11 (9, 15)	11 (8, 15)	0.75
Contrast use, median (IQR)	54 (40.5, 66)	35 (30, 50)	< 0.001

Data are presented as mean (SD), median (IQR), or *n* (%). Procedure time is defined as time from vascular access till vascular closure

*LA* left atrium

Follow-up cardiac CT was available in 128 (85.3%) of the Amulet cohort, and 124 (82.7%) of the FLX cohort. Time from LAAO to cardiac CT was 52 $\pm$ 13.9 and 53 $\pm$ 28.6 days in the Amulet and FLX cohort, respectively. Cardiac CT acquisition parameters were similar between cohorts (Table 4).

**Table 4:** Two-month cardiac CT follow-up outcomes

	Amplatzer Amulet	Watchman FLX	*p*-value
Cardiac CT available	128 (85%)	124 (83%)	0.82
Time from procedure to CT	52.8 $\pm$ 15	55 $\pm$ 34	0.39
**Acquisition parameters**			
Heart rate at CT acquisition, bpm	72.9 $\pm$ 17.4	72.8 $\pm$ 18	0.96
Systolic acquisition phase	55 (44%)	68 (55%)	0.10
Tube potential, kV	90 (90–110)	100 (90–110)	0.22
Tube current, mAs	498 (461–541)	496 (462–532)	0.36
Effective radiation exposure, mSv	1.4 $\pm$ 0.6	1.5 $\pm 0.8$	0.73
**PDL**			
Complete LAA occlusion	39 (31%)	89 (72%)	< 0.001
Grade 1 leak	24 (19%)	15 (12%)	-
Grade 2 leak	52 (41%)	NA	-
Grade 3 leak	13 (10%)	20 (16%)	-
PDL area at device disc, mm^2^	57 (24–93)	NA	-
PDL area at mid-lobe of device, mm^2^	92 (59–158)	32 (28–97)	0.019
Hypoattenuation on atrial surface of device	9 (7%)	36 (29%)	< 0.001
Definite device-related thrombus	1 (0.8%)	2 (1.3%)	0.99
Distance from LUPV to device surface, mm	0 (0–4)	11 (8–15)	< 0.001
Proximal trabeculations uncovered	5 (4%)	10 (8%)	0.13

Data are presented as mean $\pm$ SD, median (IQR), or *n* (%)

*CT* computed tomography, *LAA* left atrial appendage, *PDL* peri-device leak, *LUPV* left upper pulmonary vein

### Primary outcome analysis

The primary outcome of complete LAA occlusion was achieved in 39/128 (30.5%) patients in the Amulet cohort, and 89/124 (71.8%) in the FLX cohort, unadjusted risk ratio 2.36 (95% CI 1.77–3.1), *p* < 0.001. After adjustment for a priori defined variables (LAA landing zone size; LAA morphology; permanent AF), the risk ratio was 2.46 (95% CI 1.90–3.20), *p* < 0.001. A post hoc regression model including additional adjustment for procedural imaging guidance (ICE vs TEE), LAAO device size, and CT acquisition phase (systolic vs diastolic) yielded a risk ratio of 2.51 (95% CI 1.81–3.51), *p* < 0.001.

### Secondary outcomes of PDL

**Fig. 3:**
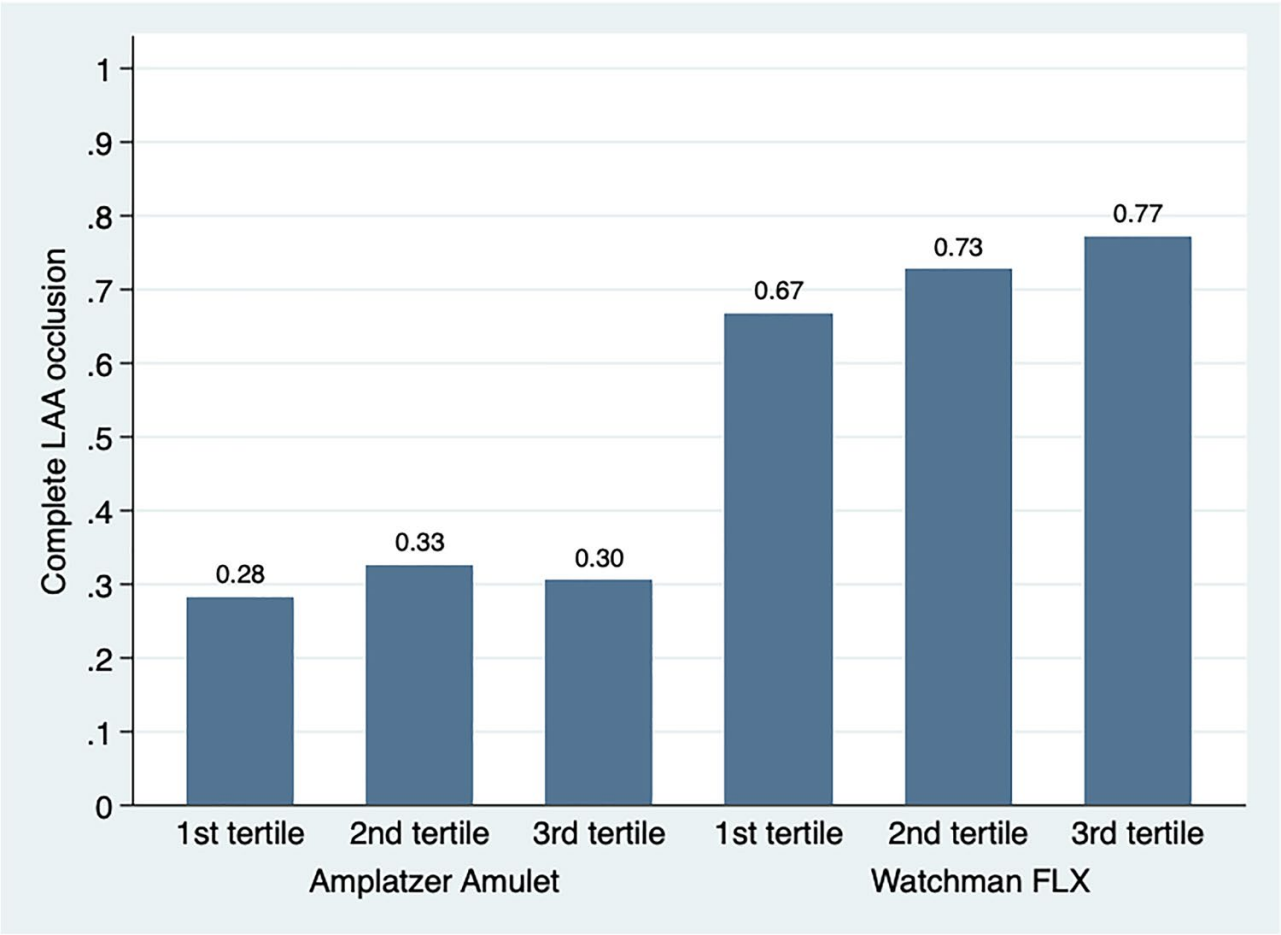
Complete LAA occlusion stratified by device cohorts into implant tertiles. Primary outcome stratified by tertiles of each device implantation cohort to assess for potential operator learning curve. The *y*-axis shows percent of patients having complete LAA occlusion defined as no visible PDL and absence of distal LAA contrast patency. LAA, left atrial appendage

**Fig. 4:**
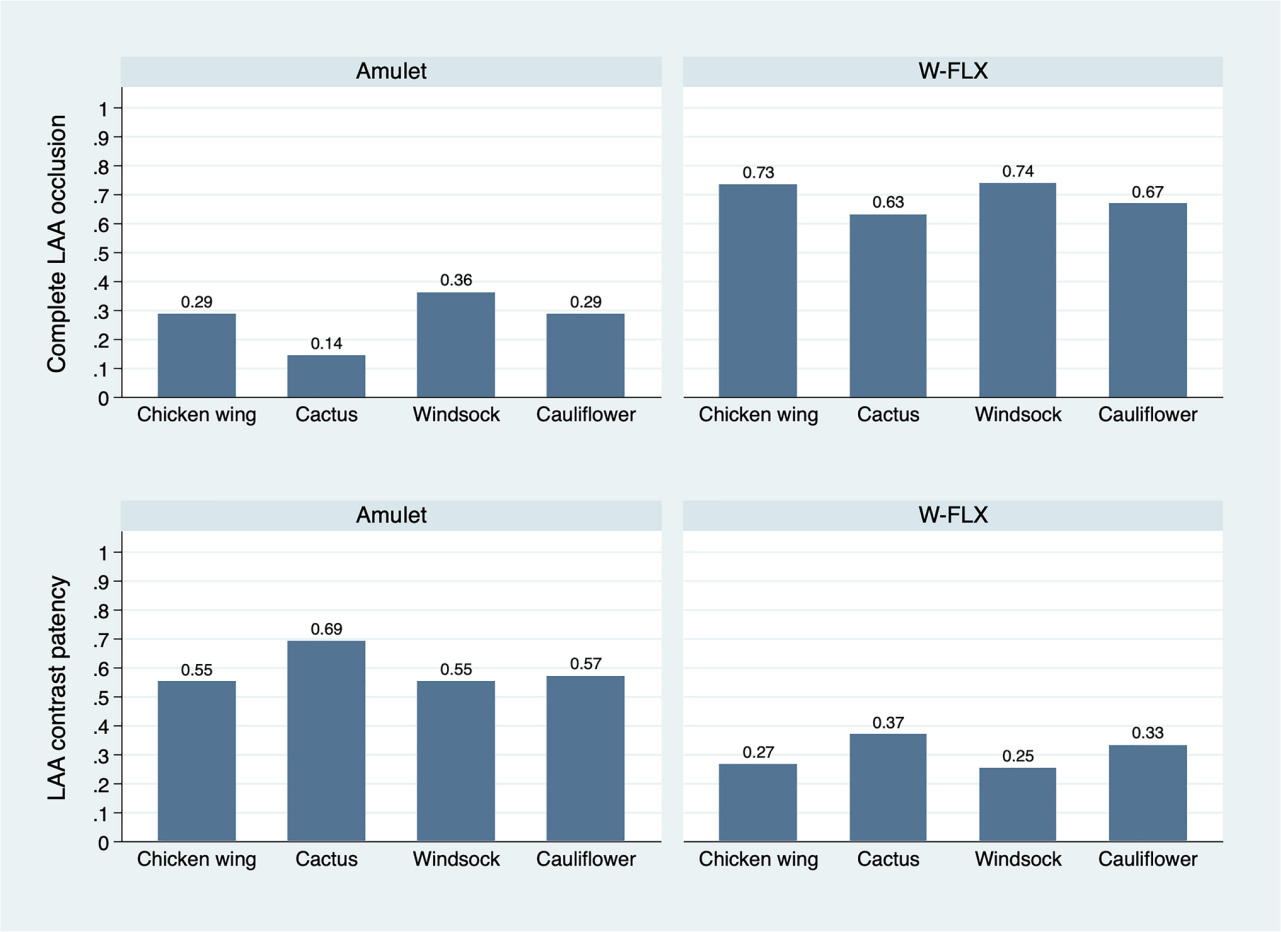
Complete occlusion and contrast patency stratified by LAA morphology. Primary outcome and contrast patency stratified by left atrial appendage morphology. LAA left atrial appendage, W-FLX Watchman FLX

In the modified analysis, considering isolated PDL at the Amulet disc without device lobe PDL or contrast patency as a complete occlusion, the LAA occlusion was achieved in 55 (43%) of the Amulet cohort, and 89 (71.8%) of the FLX cohort, with an adjusted risk ratio of 1.69 (95% CI 1.37–2.07), *p* < 0.001. Stratified analysis of each device cohort into tertiles revealed no significant sign of an operator learning curve in both the Amulet (*p* = 0.91) and FLX cohort (*p* = 0.58) as illustrated in Fig. 3. The LAA sealing results were consistent across different LAA morphologies (Fig. 4).

In the Amulet cohort, a PDL was present in 65 (50.8%) at the disc and 16 (10.7%) at the lobe. A grade 3 PDL representing PDL at both the disc and lobe and presence of contrast patency was present in 13 (10.2%) in the Amulet cohort. In the FLX cohort, a grade 3 PDL representing mid-lobe PDL and contrast patency was present in 20 (16.7%). Overall, LAA contrast patency was present in 72 (56.3%) of the Amulet cohort, and 34 (27.4%) of the FLX cohort, *p* < 0.001. Contrast patency without visible PDL (grade 1 leak) was present in 24 (18.8%) of the Amulet cohort, and 15 (12.1%) of the FLX cohort at 2 months.

The median (IQR) PDL area at the Amulet disc was 57 (24–93) mm^2^, and at the lobe 92 (59–158) mm^2^. In FLX cases, median (IQR) PDL area was 32 (28–96) mm^2^ at the mid-lobe of the FLX device. The PDL area was significantly smaller for the FLX device when compared to the mid-lobe of the Amulet (*p* = 0.019). Cardiac CT findings are summarized in Table 4.

Median (IQR) distance from the left upper pulmonary vein ridge to the atrial surface of the device was 0 (0–4) mm for the Amulet, and 11 (8–15) mm for the FLX. Uncovered proximal trabeculations were observed in 5 (3.9%) Amulet cases and 10 (8%) FLX cases (p = 0.13). Definite device-related thrombosis was detected in 1 (0.8%) and 2 (1.3%) of Amulet and FLX device implants, respectively (*p* = 0.99).

### Periprocedural complications

Major adverse event during the first 30 days post-LAAO are displayed in Tables 4 and 5. Major adverse events occurred in 6 (3.3%) and 8 (6%) patients in the Amulet and Watchman FLX cohort, respectively (*p* = 0.29). In the Amulet cohort, the adverse events comprised one delayed pericardial effusion detected after discharge and requiring acute admission and percutaneous drainage. One device embolized during index admission and required surgical intervention due to device entrapment in the mitral valve apparatus. Finally, bleeding events were gastrointestinal (*n* = 2), urogenital (*n* = 1), and severe epistaxis (*n* = 1). In the Watchman FLX cohort, two pericardial effusions occurred immediately following the intervention and were percutaneously drained. Major bleeding events were gastrointestinal (*n* = 3) and urogenital (*n* = 2).

**Table 5:** Major adverse events during 30 days postprocedural period

	Amulet *n* = 150	W-FLX *n* = 150	*p*-value
Total major adverse events	6 (4)	8 (5.3)	0.79
Pericardial effusion requiring drainage	1 (0.7)	2 (1.3)	0.99
Major bleeding	4 (2.7)	6 (4)	0.75
Device embolization	1 (0.7)	0 (0.0)	0.99

Data are presented as *n* (%)

## Discussion

The present study demonstrates a higher LAA sealing performance with the Watchman FLX device compared to the Amplatzer Amulet based on cardiac CT follow-up. The periprocedural complication risk did not differ between the two groups.

### LAA sealing performance

The Watchman FLX and Amplatzer Amulet devices are fundamentally different in design. The Amulet is built on a disc-lobe platform with a distal lobe anchoring at the neck of the LAA, and a proximal disc sealing of the (echocardiographic) orifice of the LAA. Sealing of the Amulet relies on the lobe and/or the disc. The Watchman FLX device is plug-based, and LAA sealing relies solely on the lobe which is anchored at the neck (anatomical orifice) of the LAA, approximately 10–20 mm inside the LAA measured from the left upper pulmonary vein ridge. The differences in device design makes interpretation of results difficult and reflects our choice of performing a modified primary outcome analysis where isolated PDL at the Amulet disc was disregarded if the lobe provided complete sealing. Complete occlusion was significantly higher with the FLX device for both the primary outcome analysis and the modified primary outcome analysis. This difference appeared to be driven by a higher rate of PDL at the level of the Amulet disc, whereas PDL at both the disc and lobe was less frequent than PDL with the FLX device. Distal LAA contrast patency, regardless of visible PDL or not, was significantly more common with the Amulet device and reflects incomplete LAA sealing regardless of the mechanism behind this. Both the primary outcome analysis and modified analysis also favored the FLX device, although grade 3 PDL was more common in the FLX group. Finally, the size of the PDL was significantly smaller with the FLX device. The results did not appear to be affected by operator learning curve, while they appeared consistent across various LAA morphologies. The inclusion of two separate consecutive cohorts might introduce bias favoring the latest included cohort due to a generally increasing learning curve. Nevertheless, the operators were extensively familiar with the Amplatzer devices prior to sampling of the study cohorts and had minimal experience with the Watchman devices prior to implantation of Watchman FLX. The FLX study cohort included the initial operator learning curve with this new device, which would bias the results in favor of the Amulet. The choice of intraprocedural imaging might affect the ability to detect PDL after device deployment, with the lack of multiplane and 3D capabilities currently considered a limitation of 2D-ICE-guidance. However, the current literature reports similar PDL frequency at follow-up regardless of TEE or ICE-guided interventions (13, 17, 18), and the use of ICE for procedural guidance was similar between device groups and may likely not explain the observed difference in PDL at follow-up.

The relationship between PDL (the primary outcome) and clinical events requires further study, yet the goal of LAAO is to completely seal off the LAA from the systemic circulation—hence, we should strive to achieve no contrast passage into the LAA at follow-up cardiac CT.

The Amulet-IDE trial reported a higher frequency of PDL with the Watchman 2.5 than the Amulet device, based on 45-day TEE. Only 46% of Watchman 2.5 implants were without any PDL, and the difference between devices was mainly in PDL between 3 and 5 mm of size (10). Previous studies have consistently reported around 40% PDL at 45-day TEE after Watchman 2.5 implantation (19). The PINNACLE-FLX study reported the initial experiences with the Watchman FLX device showing signs of any PDL in 17% by core-lab adjudicated 45-days TEE (9). Subsequent observational studies on Watchman FLX devices have confirmed the low rate of PDL on TEE and cardiac CT (7, 8).

Cardiac CT offers a higher sensitivity for detection of PDL, with approximately twice as many PDL reported compared to TEE (5). Cardiac CT may even be able to detect signs of incomplete device endothelialization (5, 6, 16, 20). The recent SWISS-APERO trial randomized patients between Amulet and Watchman implantation, with subsequent cardiac CT follow-up evaluation of LAA sealing. The primary outcome of LAA contrast patency was comparable between device groups and occurred in 67–70%. Visible PDL was more frequent with the Watchman device, 34 versus 22.9% (11). The Watchman group, however, included both Watchman 2.5 (23%) and FLX devices likely confounding the results. The non-randomized design of our study may introduce bias and confounding, yet the pronounced difference in primary outcome might not solely be attributed to between group differences. A canine study previously reported incomplete device endothelialization to be more common with the Amplatzer Cardiac Plug (ACP) than the Watchman 2.5 device and speculated that this was related to an observed more loose contact between the ACP disc and the LA wall as sits as a lit on the LAA orifice (21).

Nevertheless, our results are different than the LAA sealing results observed in the SWISS-APERO trial, and these results require confirmation before making solid conclusions.

### Clinical significance of PDL

Contrast patency with or without PDL may be two different clinical entities. Presence of a PDL theoretically leave the patient at risk of residual thromboembolism from the LAA. Contrast patency without PDL may reflect incomplete endothelialization and whether this associates to a residual risk of device-related thrombosis is unknown.

Despite the differences observed in LAA sealing performance in the AMULET-IDE trial, both clinical safety and effectiveness endpoints at 12 and 18 months were comparable between device groups (9). This questions the clinical significance of PDL, which is inadequately described in the current literature (5). Nevertheless, a recent study from the North American LAAO registry found a modest, but significant association between PDL (1–5 mm) and thromboembolic complications after Watchman 2.5 implantation (22). The cut-off for clinically acceptable PDL size has been arbitrarily defined and the significance remains debatable (5, 19, 23). The natural history of PDL is unclear, but smaller PDL (< 3 mm) adjacent to Watchman 2.5 devices may reduce in size over time (24), while a cardiac CT and TEE study revealed no significant PDL size reduction over time with Amplatzer devices (25). Nevertheless, contrast patency at 1 year is common and likely represents incomplete endothelialization with contrast passing through the device membrane. A higher frequency of device-related thrombosis has been reported in patients with PDL (26), which theoretically may be explained by contrast patency and PDL being a surrogate for incomplete device endothelialization. Whether contrast patency/incomplete endothelialization has clinical implications is currently unknown but should be further investigated. The higher rate of complete occlusion with the Watchman FLX device observed in our study may indicate enhanced device endothelialization, but the clinical significance hereof warrants further scrutiny.

### Clinical outcomes

The AMULET-IDE and SWISS-APERO trials both reported a significantly higher procedure-related complication rate with the Amulet compared to Watchman device, mainly driven by a difference in pericardial effusion. Our study was not designed nor powered for clinical outcomes; however, the rate of overall periprocedural complications was comparable. From both a patient and physician perspective, an embolized device resulting in surgical intervention may be weighted more severe than pericardial effusion percutaneously drained. Nevertheless, the numerical difference in device embolization and pericardial effusion between device group may likely be due to chance.

### Limitations

We acknowledge the inherent limitation of the observational study design making it susceptible to bias and confounding. By including two consecutive device series, we attempted to reduce selection bias, and the results appeared consistent in adjusted and stratified models. The single-center design limits the external validity of our results. Although imaging analysis was blinded to clinical outcomes, the interpreter can obviously not be blinded to the device implanted. The study is limited by lack of long-term imaging follow-up and clinical outcome data for the two cohorts.

## Conclusions

At 2-month follow-up, complete occlusion was achieved in a higher proportion of patients treated with the Watchman FLX compared to the Amulet device. PDL was smaller for the FLX than the Amulet. Conceptual device design differences make interpretation of the results complex and further randomized head-to-head studies with clinical outcomes are needed.

## Abbreviations

AF: Atrial fibrillation
CT: Computed tomography
ICE: Intracardiac echocardiography
LA: Left atrium
LAA: Left atrial appendage
LAAO: Left atrial appendage occlusion
PDL: Peridevice leak
TEE: Transesophageal echocardiography
