# Correction to: Left atrial appendage sealing performance of the Amplatzer Amulet and Watchman FLX device

**DOI:** 10.1007/s10840-022-01397-5

**Published:** 2022-10-21

**Authors:** Kasper Korsholm, Anders Kramer, Asger Andersen, Jacqueline Saw, Bjarne Linde Nørgaard, Jesper Møller Jensen, Jens Erik Nielsen-Kudsk

**Affiliations:** 1 Department of Cardiology, Aarhus University Hospital, Palle Juul-Jensens, Boulevard 99, 8200 Aarhus, Denmark; 2 Department of Cardiology, Vancouver General Hospital, Vancouver, Canada

**Correction to: Journal of Interventional Cardiac Electrophysiology**

10.1007/s10840-022-01336-4

The article "Left atrial appendage sealing performance of the Amplatzer Amulet and Watchman FLX device", written by Kasper Korsholm, Anders Kramer, Asger Andersen, Jacqueline Saw, Bjarne Linde Nørgaard, Jesper Møller Jensen and Jens Erik Nielsen‑Kudsk, was originally published electronically on the publisher’s internet portal on 11 August 2022 without open access. With the author(s)’ decision to opt for Open Choice the copyright of the article changed on 04 October 2022 to © The Author(s) 2022 and the article is forthwith distributed under a Creative Commons Attribution 4.0 International License, which permits use, sharing, adaptation, distribution and reproduction in any medium or format, as long as you give appropriate credit to the original author(s) and the source, provide a link to the Creative Commons licence, and indicate if changes were made. The images or other third party material in this article are included in the article’s Creative Commons licence, unless indicated otherwise in a credit line to the material. If material is not included in the article’s Creative Commons licence and your intended use is not permitted by statutory regulation or exceeds the permitted use, you will need to obtain permission directly from the copyright holder. To view a copy of this licence, visit http://creativecommons.org/licenses/by/4.0.

The original article has been corrected.

